# Intraindividual Comparison Between [^18^F] PSMA-1007 PET/CT and Multiparametric MRI for Radiotherapy Planning in Primary Prostate Cancer Patients

**DOI:** 10.3389/fonc.2022.880042

**Published:** 2022-07-14

**Authors:** Ioana M. Marinescu, Simon K. B. Spohn, Selina Kiefer, Peter Bronsert, Lara Ceci, Julius Holzschuh, August Sigle, Cordula A. Jilg, Alexander Rühle, Tanja Sprave, Nils H. Nicolay, Robert Winzer, Jana Rehm, Jörg Kotzerke, Tobias Hölscher, Anca L. Grosu, Juri Ruf, Matthias Benndorf, Constantinos Zamboglou

**Affiliations:** ^1^ Department of Radiation Oncology, Medical Center—University of Freiburg, Faculty of Medicine, University of Freiburg, Freiburg im Breisgau, Germany; ^2^ German Cancer Consortium (DKTK) Partner Site Freiburg, Freiburg im Breisgau, Germany; ^3^ Institute for Surgical Pathology, Medical Center—University of Freiburg, Faculty of Medicine, University of Freiburg, Freiburg im Breisgau, Germany; ^4^ Department of Urology, Medical Center—University of Freiburg, Faculty of Medicine, University of Freiburg, Freiburg im Breisgau, Germany; ^5^ Department of Nuclear Medicine, Faculty of Medicine, University of Dresden, Dresden, Germany; ^6^ German Cancer Consortium (DKTK), Partner Site Dresden, Dresden, Germany; ^7^ Department of Radiation Oncology, Faculty of Medicine, University of Dresden, Dresden, Germany; ^8^ Department of Nuclear Medicine, Medical Center—University of Freiburg, University of Freiburg, Faculty of Medicine, Freiburg im Breisgau, Germany; ^9^ Department of Radiology, Medical Center—University of Freiburg, University of Freiburg, Faculty of Medicine, Freiburg im Breisgau, Germany; ^10^ Tumorbank Comprehensive Cancer Center Freiburg, Medical Center—University of Freiburg, Faculty of Medicine, University of Freiburg, Freiburg im Breisgau, Germany; ^11^ German Oncology Center, European University Cyprus, Limassol, Cyprus; ^12^ Berta-Ottenstein-Programme, Faculty of Medicine, University of Freiburg, Freiburg im Breisgau, Germany

**Keywords:** positron-emission tomography, multiparametric MRI, radiation therapy, prostate cancer, PSMA

## Abstract

**Introduction:**

Accurate detection and segmentation of the intraprostatic gross tumor volume (GTV) is pivotal for radiotherapy (RT) in primary prostate cancer (PCa) since it influences focal therapy target volumes and the patients’ cT stage. The study aimed to compare the performance of multiparametric resonance imaging (mpMRI) with [^18^F] PSMA-1007 positron emission tomography (PET) for intraprostatic GTV detection as well as delineation and to evaluate their respective influence on RT concepts.

**Materials and Methods:**

In total, 93 patients from two German University Hospitals with [^18^F] PSMA-1007-PET/CT and MRI (Freiburg) or [^18^F] PSMA-1007-PET/MRI (Dresden) were retrospectively enrolled. Validated contouring techniques were applied for GTV-PET and -MRI segmentation. Absolute tumor volume and cT status were determined for each imaging method. The PCa distribution from histopathological reports based on biopsy cores and surgery specimen was used as reference in terms of laterality (unilateral *vs*. bilateral).

**Results:**

In the Freiburg cohort (*n* = 84), mpMRI and PET detected in median 2 (range: 1–5) and 3 (range: 1–8) GTVs, respectively (*p* < 0.01). The median GTV-MRI was significantly smaller than the GTV-PET, measuring 2.05 *vs*. 3.65 ml (*p* = 0.0005). PET had a statistically significant higher concordance in laterality with surgery specimen compared to mpMRI (*p* = 0.04) and biopsy (*p* < 0.01), respectively. PSMA PET led to more cT2c and cT3b stages, whereas cT3a stage was more pronounced in mpMRI. Based on the cT stage derived from mpMRI and PET information, 21 and 23 as well as 59 and 60 patients, respectively, were intermediate- and high-risk according to the National Comprehensive Cancer Network (NCCN) v1.2022 criteria. In the Dresden cohort (*n* = 9), similar results were observed.

**Conclusion:**

Intraprostatic GTV segmentation based on [18F] PSMA-1007 PET results in more and larger GTVs compared to mpMRI. This influences focal RT target volumes and cT stage definition, but not the NCCN risk group.

## Introduction

Accurate detection and segmentation of the intraprostatic gross tumor volume (GTV) is pivotal for definitive radiotherapy (RT) in patients with primary prostate cancer (PCa). First, the intraprostatic tumor volume and its extension (*e*.*g*., infiltration of the seminal vesicles) may affect the patients’ cT stage and thus the patients’ individual risk group. Consequently, it affects the RT concepts in terms of androgen deprivation therapy (ADT) administration and clinical target volumes (CTVs). Second, a precise intraprostatic GTV definition is of importance for focal RT. Interest for this has been gained in the last years since its thorough coverage is a prerequisite for successful focal RT approaches. Currently, multiparametric magnetic resonance imaging (mpMRI) is the gold standard for intraprostatic GTV detection and segmentation (1). However, previous studies suggested that mpMRI underestimates true GTV volume and misses clinically significant lesions (2–4). In parallel, positron emission tomography with prostate-specific membrane antigen (PSMA)-labeled tracers has emerged as a valuable technique for staging primary and recurrent PCa (5–8).

The current study aimed to (i) compare the performance of mpMRI with [^18^F] PSMA-1007 PET for intraprostatic GTV detection as well as delineation in patients with primary PCa and to (ii) evaluate their respective influence on RT concepts. Therefore, patients from two German university hospitals were included, and validated contouring techniques were applied for GTV segmentation (5, 9). Additionally, histology information was considered as the standard of reference by considering the PCa laterality in surgery specimen and prostate biopsy cores.

## Materials and Methods

### Patients

This study consists of patients from two university hospitals in Germany:

(1) Center 1, Freiburg: In total, 84 patients with biopsy-proven primary adenocarcinoma of the prostate who underwent [^18^F] PSMA-1007 PET/CT and 3-tesla MR imaging before any therapy (53 patients received a primary RT, 29 patients underwent open or robot-assisted prostatectomy, and three patients received androgen deprivation therapy +/- docetaxel chemotherapy) were retrospectively enrolled. The exclusion criteria were defined as any therapeutic interventions prior to imaging (such as androgen deprivation therapy or previous transurethral prostate resection) and a time difference between the PSMA PET and the MRI scan greater than 120 days. Additionally, information regarding tumor laterality was extracted based on biopsy cores and surgery specimen (unilateral *vs*. bilateral). The data was available in the form of histopathological or tumor board reports. The institutional review board of the Albert-Ludwigs-University Freiburg (Germany) approved the study (no. 476/19) (please see **Table 1** for the detailed patients’ characteristics).

**Table 1:** Patients’ characteristics.

Freiburg cohort	
Patients, *n*	84
Median age in years (range)	69.5 (49–90)
Median PSA before imaging, ng/ml (range)	11.95 (0.7–159)
Median time gap between mpMRI and PSMA-PET in days (range)	34 (0–114)
Gleason Score in biopsy cores, *n*	
6	4
7a	28
7b	26
8	16
9	8
10	1
Unknown	1
Patients with available information on biopsy cores, *n*	83
Median percent of positive biopsy cores (range)	33.33 (3.33–100)
Patients with available information on surgery specimen, *n*	28
**Dresden cohort**	
Patients, *n*	9
Median age in years (range)	68 (58–80)
Median PSA before imaging, ng/ml (range)	30.3 (6.5–126)
Gleason Score in biopsy cores	
6	0
7a	3
7b	2
8	1
9	1
10	1

(2) Center 2, Dresden: Nine patients with biopsy-confirmed primary prostate cancer who received [^18^F] 1007-PSMA PET/MRT before therapy were recruited retrospectively. Any therapeutic procedures performed before imaging were defined as exclusion criteria. The inclusion criteria were histopathologically confirmed primary prostate cancer, a pre-treatment [^18^F] 1007-PSMA PET/MRI scan, and a scheduled radical prostatectomy. Between June 2020 and October 2021, 9 patients were enrolled retrospectively (please refer to **Table 1** for the patients’ characteristics). All patients provided written informed consent.

### MR Imaging

Center 1, Freiburg: *In vivo* prostate MRI was performed with 3-tesla magnets (MAGNETOM Trio Tim, MAGNETOM Skyra, MAGNETOM Vida; all Siemens, Germany). For image acquisition, no endorectal coil was used. In all patients, at least biplanar T2-weighted imaging and diffusion-weighted imaging were performed. Additionally, a very high *b*-value image was extrapolated with *b* = 1,400 s/mm^2^ following PI-RADS recommendations. Dynamic contrast-enhanced images were acquired in the patients examined with Skyra and Vida (please see our previous publication for a more detailed information on our MR imaging protocols) (10).

### PET Imaging

Center 1, Freiburg: [^18^F] PSMA-1007 had been synthesized according to Cardinale et al. (11). The patients underwent a whole-body PET scan starting 2 h after injection (median activity in megaBecquerel: 313 MBq, range: 245–454 MBq). The scans were performed with a 64-slice Vereos PET/CT scanner in 61 patients and with a Gemini TF Big Bore in 23 patients (Philips Healthcare, USA). During the PET scan, a contrast-enhanced diagnostic CT scan (120 kVp, 100–400 mAs, dose modulation) was performed. The tracer uptake was quantified using standardized uptake values (SUV) normalized body weight.

### PET/MR Imaging

Center 2, Dresden: The preparation followed a standard protocol. 18F-PSMA was administered intravenously. The time between 18F-PSMA injection and PET/MRI was 1.5 h. PET/MRI was performed on a 3-T scanner with the patients in supine position, arms by the sides (Ingenuity TF PET/MR; Philips Health Systems, Amsterdam, Netherlands). Nine to ten bed positions with an overlap of 9 cm were acquired, with a scan time of 2 min each. The field-of-view was 18 cm, and the reconstructed isotropic spatial resolution was 5.5 mm. From the head to the distal femur (integrated quadrature body coil), low-resolution T1-weighted fast-field-echo images were acquired to create a map for attenuation correction *via* segmentation into three tissue classes (air, lung, and soft tissue), followed by the assignment of respective attenuation values. The patient’s position was maintained throughout the procedure to ensure optimal co-registration, and PET was performed immediately following the attenuation scan. MRI was performed according to the treatment center’s standard protocol for pelvic MRI in the follow-up of pelvic malignancy. Thus, all pelvic MRIs included T2-weighted, diffusion-weighted, and T1-weighted contrast-enhanced sequences (Sense-Xl-Torso coil). Apparent diffusion coefficient (ADC) maps were generated automatically. Contrast-enhanced sequences were performed about 60 s after the intravenous administration of 0.2 ml gadolinium diethylenetriamine penta-acetic acid or 0.1 ml gadobutrol per kilogram of body weight (Magnevist^®^/Gadovist^®^, Bayer Pharma, Berlin, Germany), followed by 20 ml saline. Philips Fusion Viewer software was used to create merged PET/MR images, including multiplanar reconstructions.

### Image Delineation

One board-certified radiologist (MB) and one board-certified radiation oncologist (CZ) with >6 years of experience in interpretation of prostate MRI delineated all areas suspicious for a clinically significant tumor in the axial T2w sequences (GTV-MRI) in consensus. For delineation of T2w images, DWI (including the extrapolated *b*-value image) and ADC maps were available. Standardized imaging criteria (PI-RADSv2.1) were applied for tumor delineation. Lesions with a PI-RADS category ≥3 were considered positive.

Two radiation oncologists with 6 (CZ) and 1–3 years (MM or SP) of experience in interpretation of PSMA-PET images, respectively, contoured GTV-PET in consensus by applying a PET image windowing from SUVmin–max 0–10 (9) in Eclipse v15.1 software (Varian Medical Systems, USA). The presence of PCa on PET images was defined as mono- or multifocal uptake greater than the adjacent background in more than one slice (GTV-PET) (9). Apart from PET and CT images for anatomical orientation, no additional clinical information was provided.

The prostate volume on CT and MR images was delineated by an experienced reader (CZ) according to the ESTRO-ACROP guidelines (12).

### Statistical Analysis

The statistical analysis was performed on GraphPad Prism v8.4.2 (GraphPad Software, USA). Normal distribution was tested using the D’Agostino and Pearson normality test. A Wilcoxon matched-pairs signed rank test was used to compare not normally distributed metric variables. For normally distributed metric variables, a paired *t*-test was used for comparison. For categorical variables, one-sided Fisher’s exact test was used. The significance level was defined as 0.05 (the figures were created on GraphPad Prism v8.4.2, GraphPad Software, USA).

## Results

### Freiburg Cohort

**Figure 1:**
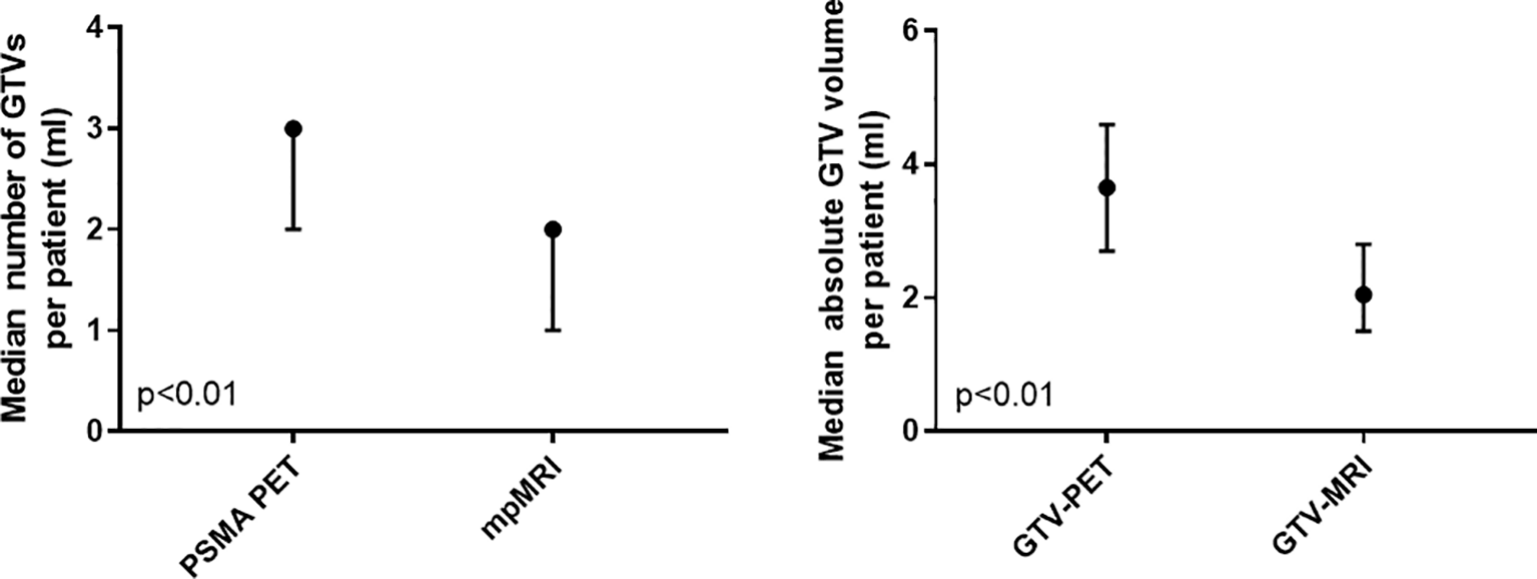
Number and absolute gross tumor volumes in mpMRI and PSMA PET on a patient basis in the entire cohort (*n* = 84). The median value and the 95% confidence interval are represented. PSMA PET, prostate-specific membrane antigen positron-emissions tomography; mpMRI: multiparametric magnetic resonance imaging.

**Figure 2:**
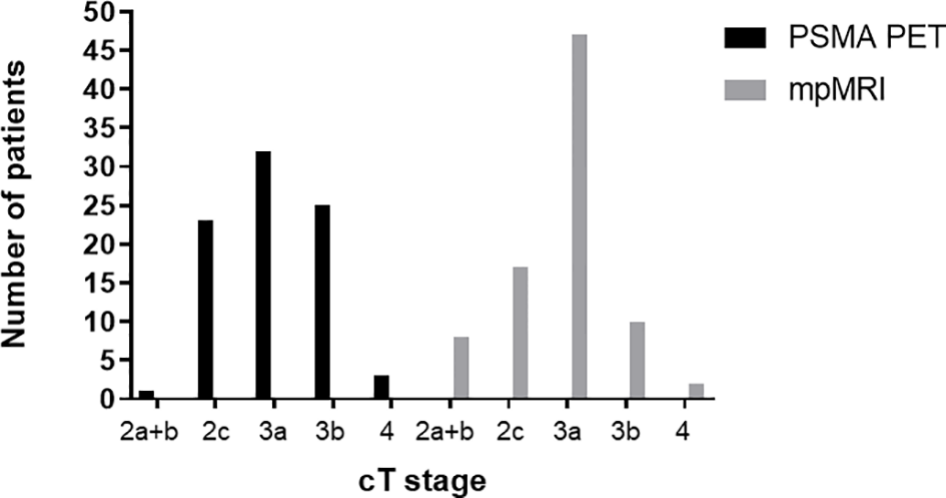
Comparison of cT stage based on PSMA PET and mpMRI in the entire cohort (*n* = 84). PSMA PET, prostate-specific membrane antigen positron-emissions tomography; mpMRI, multiparametric magnetic resonance imaging.

In the entire cohort (*n* = 84), 144 and 245 intraprostatic GTVs were detected by mpMRI and PET, respectively. On a patient basis, mpMRI and PET detected in median 2 (range: 1–5) and 3 (range: 1–8) GTVs, respectively (*p* < 0.01) (**Figure 1**). The median volume of GTV-MRI and GTV-PET per patient was 2.1 ml (range: 0.2–42.8) and 3.7 ml (range: 0.4–85), respectively (*p* < 0.01) (**Figure 1**). The distribution of the cT stages based on mpMRI and PSMA PET is represented in **Figure 2**. PSMA PET led to more cT2c (23 *vs*. 17 patients) and cT3b (25 *vs*. 10 patients) stages, whereas cT3a stage was more pronounced in mpMRI (47 vs. 32 patients). Based on the cT stage derived from mpMRI and PET information, 4 (5%) and 1 (1%), 21 (25%) and 23 (27%), and 59 (70%) and 60 (71%) patients were of low, intermediate, and high risk according to the national comprehensive cancer network (NCCN) v1.2022 criteria.

**Figure 3:**
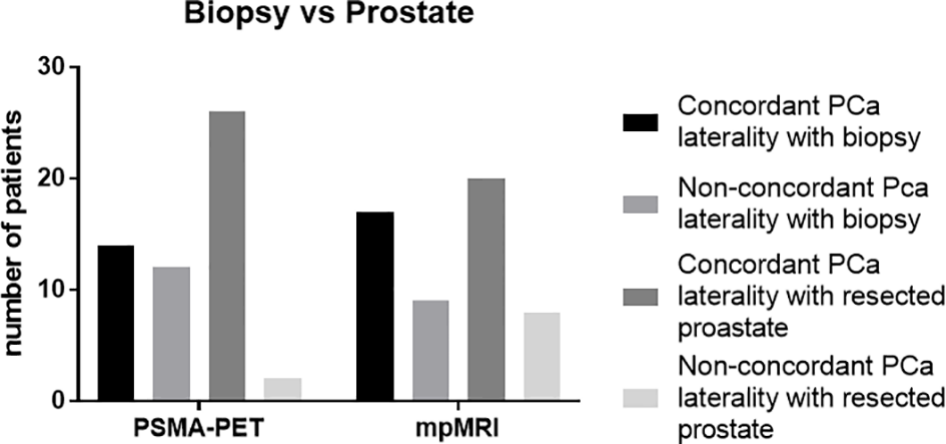
Concordance of prostate cancer laterality with biopsy (*n* = 64) and resected prostate (*n* = 28), respectively. The absolute numbers of patients are represented. PSMA PET, prostate-specific membrane antigen positron-emission tomography; mpMRI, multiparametric magnetic resonance imaging.

PCa distribution from histopathological reports based on biopsy cores and surgery specimen was available in 64 and 28 patients, respectively. First, PCa laterality on biopsy cores was considered: MRI and PSMA showed concordance in 46 patients (66%) and 44 patients (63%), respectively (*p* = 0.86). In 10 patients, solely MR imaging was concordant in PCa laterality with biopsy cores, whereas PET was not. On the contrary, PET was concordant in 8 patients, in which MRI was not. Considering the combined PET and MR information, 36 patients (51%) had concordance in laterality with the biopsy specimen. In the subgroup of patients with bilateral PCa lesions according to biopsy samples, the following were observed: MRI and PET were concordant with biopsy cores in 37 (82%) and 42 (93%) patients, respectively (*p* < 0.01). In this case, MRI was coincident with the biopsy cores in 1 patient, where PSMA was not and PSMA in 6 patients, where MRI was not (**Figure 3**).

**Figure 4:**
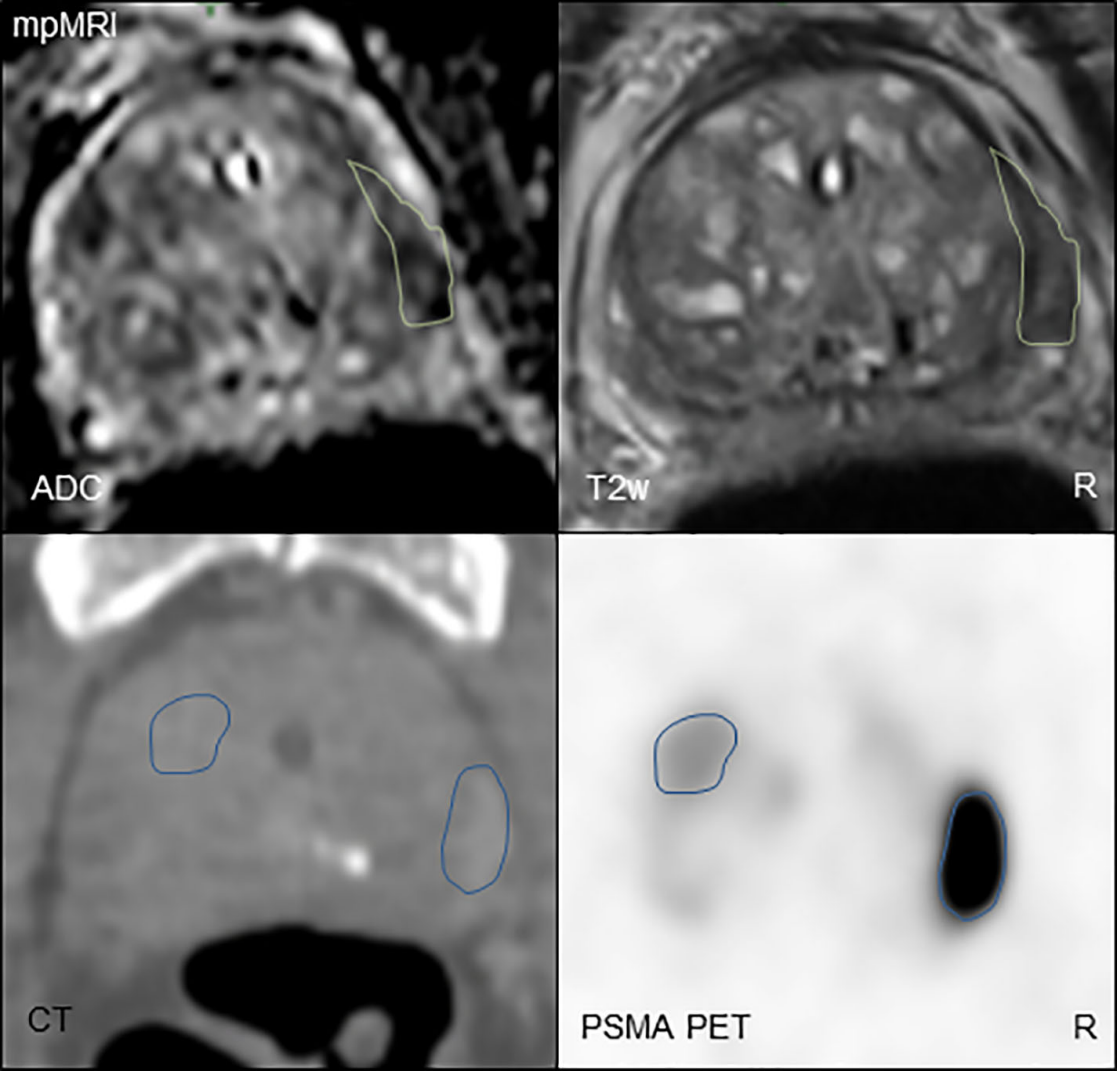
Comparison of mpMRI (above) and PSMA PET (below) imaging in a PCa patient. For mpMRI, the biplanar T2-weighted imaging and diffusion-weighted imaging are shown. For the PSMA PET (PET image windowing: SUV 0–10), the CT scan is shown for anatomical orientation. The GTVs are displayed in green (GTV-MRI) and blue (PSMA PET), respectively. GTV-MRI was smaller than GTV-PET with 1.3 and 4.9 ml, respectively. mpMRI was concordant in PCa laterality with biopsy cores (unilateral, right), whereas PSMA PET showed concordance with surgery specimen (bilateral). T2w, biplanar T2-weighted imaging; ADC, apparent diffusion coefficient; GTV, gross tumor volume; PCa, prostate cancer; PSMA PET, prostate-specific membrane antigen positron-emission tomography; mpMRI, multiparametric magnetic resonance imaging; R, right.

Second, the PCa laterality on surgery specimen was analyzed, and mpMRI and PET were in 20 (71%) and 26 (93%) of patients, congruent with the surgery specimen, respectively. PET had a statistically significant higher concordance in laterality with surgery specimen compared to mpMRI (*p* = 0.04). Third, the pT stage in surgery specimen was compared with cT stage based on PET and mpMRI, respectively. The cT stage based on mpMRI and PET was concordant with pT stage in 16 (57%) and 18 (64%) patients, respectively. **Figure 4** illustrates an example of the discordant findings between MR and PSMA PET.

### Dresden Cohort

The median volume of GTV-MRI and GTV-PET per patient was 3.8 ml (range: 0.1–59.5) and 4.4 ml (range: 2.1-60.1), respectively (*p* = 0.02). According to mpMRI and PET, 1 and 3 patients had cT3b stage, and 4 and 3 patients had cT3a stage, respectively. Five and 6 patients were high-risk according to the NCCN criteria v1.2022 in mpMRI and PET, respectively.

### Pooled Database

Finally, the pooled data from all patients (*n* = 93) was analyzed. The median volume of GTV-MRI and GTV-PET was 2.2 ml (range: 0.1–59.5) and 3.7 ml (range: 0.4–85), respectively (*p* < 0.0001). According to mpMRI and PET, 9 and 1 patients had cT2a stage, 20 and 26 had cT2c stage, 51 and 35 patients had cT3a stage, and 11 and 28 patients had cT3b stage, respectively (**Table 2**).

**Table 2:** Pooled data of all patients.

All patients	Median/*n*		*P*-value
	mpMRI	PSMA PET	
**GTV (ml)**	2.2	3.7	<0.0001
**cT** T2a T2b T2c T3a T3b T4	9 0 20 51 11 0	1 0 26 35 28 0	<0.0001

## Discussion

Several studies compared [^68^Ga]-labeled PSMA tracers with the current standard-of-care mpMRI for intraprostatic GTV detection (13–15) and segmentation (5, 16, 17) by using histopathology derived from biopsy cores or from surgery specimen as the standard of reference. All studies concluded that [^68^Ga]-PSMA PET imaging provides complementary information. However, our group reported that visual [^68^Ga]-PSMA-11 PET image interpretation misses clinically relevant PCa in terms of microscopic lesions with ISUP grade >1 in approximately 30% of patients (18). Furthermore, in their study, Kuten et al. performed a head-to-head comparison between [^18^F] PSMA-1007 and [^68^Ga] PSMA-11 PET/CT and reported similar results for both tracers, with better performance for the [^18^F] PSMA-1007 tracer in less intense foci (19). The prospective ProStaPET study compared [^18^F] DCFPyL PET and mpMRI for PCa detection by using histopathology after surgery as reference (20). The authors concluded that combined PET/MR information does not outperform mpMRI information alone. However, no dedicated segmentation process was performed in the latter trial. Consequently, we initiated this study to compare [18F] PSMA-1007 PET/CT with mpMRI for intraprostatic GTV detection and delineation using validated segmentation techniques and histopathology as the standard of reference in a subgroup of patients.

The first aim of our study was to analyze the value of [^18^F] PSMA-1007 PET and mpMRI for focal RT approaches. Focal RT can be applied in three ways: ultra-focal (RT of the PCa lesion), on a half-gland basis [RT of the gland including PCa lesion(s)], and localized (RT of the entire prostate with dose escalation to the PCa lesion(s) (21). A high-dose coverage of the intraprostatic GTV is warranted for all focal RT approaches to increase the tumor control probability (22). In the Freiburg cohort, PET detected significantly more intraprostatic PCa lesions with a significantly larger volume than mpMRI. The PET-derived GTV was also significantly larger in an external cohort. These findings are in concordance with previous studies which compared [^68^Ga]-labeled PSMA tracers with mpMRI (13–15). Taken together, the findings of our study suggest that [^18^F] PSMA-1007 is a promising tool for focal therapy guidance to intraprostatic GTVs and might outperform the stand-alone MRI. However, mpMRI should not be omitted in this context since it provides complementary information in some of the patients since no PSMA expression was reported in approximately 10% of intraprostatic GTVs (23, 24). In line with this, in our study, MR showed concordance with biopsy PCa distribution in 12% of patients in which PET failed to detect the correct distribution. In addition, it is a useful tool to delineate the prostate and the urethra during focal RT planning (25). One must consider that the implementation of PSMA PET imaging leads to larger RT volumes and a decrease in specificity. Parts of non-PCa tissue within the prostate might likewise be irradiated, which might lead to an increased risk for toxicity. Whether a RT dose escalation on GTVs based on combined PSMA PET and mpMR information is safe and increases the tumor control will be examined by the randomized controlled HypoFocal-SBRT trial in the future (26). Interestingly, PET had even a higher concordance in laterality of PCa lesion with surgery specimen compared to mpMRI and biopsy cores. This finding is crucial for half-gland RT approaches (27), as it suggests that PSMA PET should also be incorporated in this clinical scenario to decrease the risk of false-negative findings in mpMRI and biopsies.

The second aim was to compare both imaging modalities for cT stage definition in primary PCa patients. Currently, the cT stage is determined by digital rectal examination (DRE). The cT stage impacts the patients’ NCCN risk groups and consequently affects treatment concepts in terms of CTV margins (12) and ADT administration (28). In both study cohorts, PSMA PET detected more bilateral lesions and more seminal vesicle involvement than mpMRI. Seminal vesicle involvement in PET may influence RT margins in terms of a cephalad expansion of the CTV margins. In contrast, more patients had an extracapsular extension in mpMR images. The latter result is not surprising since a proper evaluation of the prostatic capsule is difficult in PET/CT due to the low soft tissue contrast of CT imaging. However, this finding might also affect RT margins by expansion of the CTV in the area of the extracapsular extension. Privé et al. compared [^18^F] PSMA-1007 PET with mpMRI by using histopathological outcome in surgery specimen as the standard of reference in 23 patients and observed comparable results (29). Nevertheless, the resulting NCCN risk groups of the patients in our study were comparable between mpMRI and PSMA. Thus, ADT concepts may not be affected by the usage of additional PET imaging. cT stage based on PET or mpMRI had only moderate concordance (57–64% of patients) with pT stage in a surgery specimen. Future studies should assess whether cT stage based on mpMRI and PSMA PET outperforms the cT stage based on DRE in terms of prognostic value for primary PCa patients.

In the following, we want to discuss the limitations of our study. First, since PET/CT scans are mainly conducted for primary PCa patients with advanced disease status, our study cohort consists primarily of patients with intermediate- and high-risk PCa. Thus, it is unclear whether our findings can be translated to low-risk PCa patients. However, low-risk patients are also suitable for ultra-focal therapies as an alternative to active surveillance. Therefore, the accuracy of PSMA-PET should be further evaluated in this patient group.

Second, the retrospective design of the study represents another limitation. Especially in the Freiburg cohort, histopathological reports from biopsy cores were available in only 64 patients (76%). Moreover, histopathology information from a surgery specimen was only available in 28 patients (33%), and histopathology information was not registered with the PET and mpMR information as was done in previous studies (4, 10). Thus, the comparison with standard-of-reference PCa in histopathology was performed on a laterality level. In the Dresden cohort, no reports were available.

Third, in particular for MR-based intraprostatic GTV contouring, an interobserver heterogeneity was reported (9,). To tackle this issue, this study implemented consensus contours from two experienced readers.

Finally, in the Freiburg cohort PET/CT and mpMRI scans were not acquired simultaneously with a median time gap between both examinations of 34 days. We addressed this issue by implementing an external cohort of patients with hybrid [^18^F] PSMA-1007 PET/MR examinations. In this context, it should be mentioned that slightly different post-injection times were used in both centers (Freiburg: 2 h and Dresden: 1.5 h) which might hamper comparability.

Given the limitations of the research in our study and other studies (18) and considering the different concordance of both imaging methods with the histologic reference, ultra-focal radiotherapy targeting solely the PCa lesion should be further investigated in controlled clinical studies.

## Conclusion

In this study, we performed an intraindividual comparison between [18F] PSMA-1007 PET/CT and mpMRI in a large cohort of patients in two different university hospitals in Germany. Using validated contouring approaches, [18F] PSMA-1007 PET showed more and larger intaprostatic tumor lesions than MRI and detected more cT2c and cT3b stages. Additionally, PET had a statistically significant higher concordance in laterality with a surgery specimen compared to mpMRI (*p* = 0.04) and biopsy (*p* < 0.01), respectively. However, MRI identified more cT3a stages and provided complementary information in >10% of patients concerning PCa localization. These findings have an effect on the volume of focal RT targets and the definition of cT stages, but not on the NCCN risk group. Consequently, both image modalities should be used for the RT planning process on primary PCa patients. Prospective trials are currently ongoing to evaluate the safety and therapeutic efficacy of focal RT using combined PSMA PET and mpMRI data.

## Data Availability Statement

The original contributions presented in the study are included in the article/supplementary material. Further inquiries can be directed to the corresponding author.

## Ethics Statement

The studies involving human participants were reviewed and approved by the institutional review board of the Albert-Ludwigs-University Freiburg (Germany) (no. 476/19). Written informed consent for participation was not required for this study in accordance with the national legislation and the institutional requirements. Written informed consent was not obtained from the individual(s) for the publication of any potentially identifiable images or data included in this article.

## Author Contributions

Conceptualization, methodology: CZ, IMM, ALG, JRuf, MB. Patient recruitment: IMM, SKBS, CZ, AS, SK, PB, AS, CAJ. Data curation: CZ, ALG, SKBS. Writing—original draft preparation: CZ, IMM. Writing—review and editing, all, Supervision: ALG, JRuf, MB. Project administration: CZ. Visualization: JH, LC, AR, TS, NHN, RW, JRehm, JK, TH. All authors have read and agreed to the published version of the manuscript.

## Funding

This study was part of the ERA PerMed JTC2018 project “PersoProCaRisk”.

## Conflict of Interest

CZ received funding from the German Cancer Consortium (DKTK), Naslund Medical, and the Klaus Tschira foundation, as well as honoraria from Johnson and Johnson and Novocure, outside the submitted work.

The remaining authors declare that the research was conducted in the absence of any commercial or financial relationships that could be construed as a potential conflict of interest.

## Publisher’s Note

All claims expressed in this article are solely those of the authors and do not necessarily represent those of their affiliated organizations, or those of the publisher, the editors and the reviewers. Any product that may be evaluated in this article, or claim that may be made by its manufacturer, is not guaranteed or endorsed by the publisher.

## Abbreviation

T2w, biplanar T2 weighted imaging; ADC, Apparent diffusion coefficient;GTV, gross tumour volume; PCa, prostate cancer; PSMA PET, prostate-specific membrane antigen positron-emission tomography; mpMRI, multiparametric magnetic resonance imaging R, right. T2w, biplanar T2 weighted imaging; ADC, Apparent diffusion coefficient;GTV, gross tumour volume; PCa, prostate cancer; PSMA PET, prostate-specific membrane antigen positron-emission tomography; mpMRI, multiparametric magnetic resonance imaging R, right.
